# Dietary amino acid composition and elevated fasting glucose in children and adolescents from four Chinese provinces: a cross-sectional study combining regression and machine learning

**DOI:** 10.3389/fpubh.2026.1912084

**Published:** 2026-10-26

**Authors:** Yao Chen, Yanfeng Shang, Hongwei Wang, Qianrang Zhu, Yiya Liu, Meina Tian, Changqing Liu, Yanqi Sun, Zhenchuang Tang, Lianlong Yu

**Affiliations:** 1 Clinical Nutrition Department, Rizhao People's Hospital, Rizhao, China; 2 Rizhao Jinhai'an Primary School, Rizhao, China; 3 Geriatrics/Health Care Department, Rizhao People's Hospital, Rizhao, China; 4 Jiangsu Center for Disease Control and Prevention, Nanjing, China; 5 Guizhou Center for Disease Control and Prevention, Guiyang, China; 6 Hebei Center for Disease Control and Prevention, Shijiazhuang, China; 7 Public Health Department, Rizhao People's Hospital, Rizhao, China; 8 Institute of Food and Nutrition Development, Ministry of Agriculture and Rural Affairs, Beijing, China; 9 Shandong Center for Disease Control and Prevention, Jinan, China; 10 School of Public Health, Shandong First Medical University, Jinan, China; 11 School of Public Health, Shandong Second Medical University, Weifang, China

**Keywords:** adolescents, children, dietary amino acids, elevated fasting glucose, machine learning

## Abstract

**Objective:**

Using an integrated analytical pipeline that combines regression and machine learning, with nonlinear dose–response modeling, this cross-sectional study sought to examine dietary amino acid composition in relation to elevated fasting glucose (EFG) in children and adolescents.

**Methods:**

Data from 12,567 children and adolescents (6–18 years) from four provinces in a national nutrition and health surveillance program were analyzed. Feature selection was performed using the Boruta algorithm, and propensity score matching (PSM) was utilized to address confounding. Conditional logistic regression was used to assess the relationships between EFG risk and 20 amino acids expressed as percentages of total protein intake; odds ratios (ORs) were reported per 1-percentage-point increment in the proportion of total protein intake. Predictive models were built with six machine learning algorithms (LightGBM, XGBoost, SVM, KNN, CatBoost, and NB) and the SHAP method was used to measure feature contributions and determine the most important amino acids. Further stratified analyses and a generalized additive model (GAM) were conducted to assess association strength and investigate possible nonlinear dose–response relationships.

**Results:**

6,960 participants were included in the final analysis. The best-performing model was LightGBM (AUC = 0.831), which included this model included 20 amino acids plus 12 confounding factors (including fat intake, protein intake, hypertension, and general/central obesity). Conditional logistic regression combined with SHAP-based feature importance ranking identified methionine (OR = 2.03, 95% CI: 1.61–2.57) and threonine (OR = 1.59, 95% CI: 1.32–1.92) as positively associated with EFG, whereas arginine (OR = 0.82, 95% CI: 0.74–0.92) and tyrosine (OR = 0.71, 95% CI: 0.59–0.86) were inversely associated (all *p* < 0.01). Stratified analyses showed generally consistent directions of association. The GAM showed that methionine, arginine, and tyrosine had significant nonlinear relationships with EFG risk (*P* for nonlinearity < 0.05).

**Conclusion:**

The dietary amino acid intake is strongly associated with EFG levels in children and adolescents. Methionine and threonine are positively associated, and arginine and tyrosine are inversely associated. Collectively, these four amino acids may be important dietary correlates associated with abnormal glucose metabolism in this study population.

## 1 Introduction

As socioeconomic development and dietary patterns change in China, the trend of nutrition-related chronic diseases in children and adolescents has been on the rise, which is also evident in other parts of the world. Among them, elevated fasting glucose (EFG) has become a significant public health issue that has impacted the health of children and adolescents, especially in China (1–3). Research has demonstrated that childhood and adolescent abnormal glucose metabolism not only retards growth and development, but also greatly predisposes the individual to chronic diseases like diabetes and cardiovascular disease in adulthood (4–6). Thus, determining the most important risk factors of EFG in children and adolescents, especially modifiable dietary factors, is of significant clinical and population health value in creating specific prevention measures and reducing the tendency toward the early onset of diabetes.

One of the factors that regulate glucose metabolism is dietary nutrition. Proteins are essential nutrients that are digested and absorbed into amino acids, which directly or indirectly affect glucose homeostasis by being involved in various physiological processes, such as insulin sensitivity regulation, insulin secretion, and energy metabolism (7–9). As an illustration, certain subgroups of amino acids, including aromatic amino acids (AAA), sulfur-containing amino acids (SAA), and branched-chain amino acids (BCAA) have been found to be strongly linked with the risk of type 2 diabetes (T2DM) in adults (9–13). Nevertheless, children and adolescents are at a critical stage of growth and development, and their metabolic features of amino acids and dietary intake patterns are significantly different compared to adults (14). The current literature on the relationship between dietary amino acids and EFG in this population is mostly extrapolated based on adult evidence, and there is a significant gap in the literature on the relationship between dietary amino acids and EFG in children, especially large-scale evidence from China. Consequently, it is not clear what amino acids are important dietary predictors of high blood glucose among Chinese children and adolescents.

In addition, the past research has mostly been based on the conventional statistical techniques, which can be weak in determining the core relationships in high-dimensional dietary data and can be affected by multicollinearity and intricate interrelationships among variables. Conversely, machine learning methods, with their high nonlinear modeling ability and high-dimensional feature selection benefits, can better remove irrelevant variables and measure the relative importance of each amino acid, thus enhancing the accuracy and specificity of key feature identification (15–19). Building on the analytical framework established in our previous pediatric metabolic studies (18, 19), this study applied comparable dietary amino acid measures and machine learning tools to examine factors associated with EFG, a metabolic endpoint that remains understudied in Chinese children and adolescents. Using large-sample data from four provinces, we systematically identified key amino acids associated with EFG and further examined the direction and strength of these associations. The results are scientifically supported to develop individualized and specific nutritional prevention measures to decrease the risk of abnormal glucose metabolism in children and adolescents by amino acid-based nutritional control, and address a research gap in this area in China.

## 2 Methods

### 2.1 Study population and data source

The data used in this study were based on the Chinese National Nutrition and Health Surveillance of Children and Lactating Mothers (2016–2019), which is a large national public health surveillance project organized by the National Institute of Nutrition and Health (NINH), Chinese Center of Disease Control and Prevention (China CDC). The objective of the project was to evaluate the health and nutritional status, dietary intake of foods and nutrients, and other factors that influence them in children and lactating mothers in China in a systematic manner.

The parent surveillance used a multistage stratified cluster random sampling design with survey weights. A total of 125 surveillance sites were selected across 31 provinces, covering four types of regions: large cities, small and medium-sized cities, general rural regions, and impoverished rural regions. This study used surveillance data of four provinces (Shandong, Jiangsu, Hebei, and Guizhou) that comprised both urban and rural sites, and thus, provided a wide representativeness of the national population of children and adolescents aged 6–18 years, based on the principles of comprehensive coverage of geographic regions, urban–rural development levels, and dietary patterns. Multidimensional data were gathered by using interviews, physical examination, laboratory tests, and standardized dietary assessment. The Ethics Committee of the China CDC approved this study (Approval No. 201614), and all participants or their legal guardians gave written informed consent.

There were 23,303 participants initially. We excluded sequentially individuals aged out of 6–18 years (*n* = 10,347), those with missing height or weight data (*n* = 3), those who did not complete the dietary survey or had missing dietary data (*n* = 124), and those with energy intake that was not within the 1st and 99th percentiles of the same age- and sex-specific distribution (*n* = 262). Following the use of these criteria, 12,567 participants were incorporated in the final analysis.

### 2.2 Data collection

#### 2.2.1 Questionnaire survey

The information about demographic features (age, sex, and school type), lifestyle (e.g., physical activity, screen time), and personal and family history of diseases was collected using a standardized electronic questionnaire that was administered in face-to-face interviews. Moderate-to-vigorous intensity physical activity (MVPA) was considered as any type of activity that causes rapid breathing or sweating, including running, cycling, swimming, sporting activities, or household chores.

#### 2.2.2 Physical measurements

All physical measurements were performed by trained investigators in the morning under fasting conditions. The anthropometric measurements were performed according to the Chinese Health Industry Standard (WS/T424-2013) (20). An electronic sphygmomanometer was used to measure blood pressure three times on the right upper arm and the average was taken. The Chinese National Health Industry Standard (WS/T610-2018) (21) defined hypertension as a systolic and/or diastolic blood pressure that is 95th percentile or higher of age-, sex-, and height-specific reference values. General obesity was determined using the age- and sex-specific body mass index (BMI) standard (WS/T 586–2018) (22). The definition of central obesity was based on the waist circumference (WC) percentile, i.e., a waist circumference of 90th percentile in relation to the age and sex (23, 24).

#### 2.2.3 Laboratory testing

The participants were required to fast for 8–12 h following dinner on the eve before blood collection. The next morning, 6 mL of fasting venous blood was taken out of the cubital vein into serum separator tubes with clot activator. Samples were left to stand at room temperature for 30 min and centrifuged at 3,000 rpm for 10 min to separate serum. The serum was aliquoted and stored at −80 °C until analysis.

The hexokinase method was used to measure fasting plasma glucose (FPG) on an automated biochemical analyzer. Each analytical batch contained high- and low-concentration quality control samples to provide accuracy and reliability. The 2017 American Diabetes Association (ADA) diagnostic criteria defined elevated fasting glucose (EFG) as FPG ≥ 5.6 mmol/L, including impaired fasting glucose (IFG, 5.6–6.9 mmol/L) and diabetes (FPG ≥ 7.0 mmol/L) (25).

#### 2.2.4 Food intake assessment

A food frequency questionnaire (FFQ) was used to measure dietary intake. Face-to-face interviews were used to gather data on the frequency and average quantity of different foods eaten by the participants in the last month by trained investigators. Food items were classified into 12 food groups and 59 subcategories. The frequency of consumption and the portion size of each item were recorded, and food models and food atlases were utilized to help the participants estimate the weights of portions. This FFQ had been extensively applied and tested in a number of large-scale nutritional studies of children in China (18, 26–29).

A consecutive 3-day (2 school days + 1 weekend day) weighing technique was used to determine the average daily intake of cooking oil and major condiments in the households of participants (or school cafeterias) to estimate the intake of these products, and these household- or canteen-level amounts were then allocated to individual participants in proportion to their daily energy intake estimated from the FFQ. The Chinese Food Composition Table (30) was used to calculate daily energy and macronutrient intake. The intake of amino acids was estimated using the FFQ data and the amino acid composition database of the food composition table. The nutrient density approach was used to reduce multicollinearity due to the high correlation between amino acid intake and total protein intake (18, 31–35), with the proportion of each amino acid relative to total-protein intake as the main analytical variable.

### 2.3 Covariate definitions

The covariates in this study were demographic, physical activity, health status, and dietary variables. Demographic factors were sex (male/female), age (continuous), and school type (primary/secondary school). The variables of physical activity were MVPA status (achieved/not achieved) and MVPA duration (continuous). The variables of health status were hypertension (yes/no), general obesity (yes/no), and central obesity (yes/no). The dietary variables were total energy intake (continuous), protein intake (continuous), carbohydrate intake (continuous), and fat intake (continuous). Variance inflation factors (VIFs) were determined before the construction of the models to determine the presence of multicollinearity among covariates. The findings showed that there were a high collinearity between total energy intake and some of the variables related to macronutrients. Because amino acid exposure was expressed as a percentage of total protein intake, total protein intake was highly correlated with the amino acid variables. In order to reduce model instability and possible estimation bias, and to guarantee the robustness and reliability of the statistical analyses, protein intake, fat intake, and carbohydrate intake were not included in the final list of confounding variables.

### 2.4 Statistical analysis

Initially, propensity score matching (PSM) was conducted to address the possibility of confounding bias, with a matching ratio of 1:3 of the EFG group to the normal control group. Logistic regression was used to estimate propensity scores with EFG status as the dependent variable and age, sex, and MVPA duration as covariates. Nearest-neighbor matching was used with a caliper width of 0.025.

Boruta model was used to rank feature importance, including sociodemographic characteristics (sex, age, and school type), physical activity (MVPA status and duration), hypertension, general obesity, central obesity, total dietary energy intake, three macronutrient intakes, and the intake levels of 20 dietary amino acids to identify key amino acid features associated with EFG risk.

The Shapiro–Wilk test was used to test the normality of the continuous variables, and the results showed that not all the variables were normally distributed (*p* < 0.05). Therefore, continuous variables were presented as median (Q₁, Q₃), and between-group comparisons used the Mann–Whitney *U* test. Categorical variables are represented as frequencies (percentages), and the difference in groups was evaluated with the help of the *χ*^2^ test.

Correlation analysis between EFG and 20 dietary amino acids was performed in the overall population, as well as stratified by male and female subgroups. Correlation heatmaps were generated to visually display the correlation magnitude and direction.

Conditional logistic regression was used to evaluate associations between EFG and the 20 amino acids. Three increasingly adjusted models were built: Model 1, unadjusted; Model 2, adjusted for school type and total energy intake; and Model 3, further adjusted by general obesity, central obesity, and hypertension on the basis of Model 2. E-values with 95% confidence intervals were simultaneously calculated based on Model 3 to assess the robustness of the observed associations against unmeasured confounding.

The predictive models of EFG risk were built with six machine learning algorithms: Light Gradient Boosting Machine (LightGBM), Extreme Gradient Boosting (XGBoost), support vector machine (SVM), k-nearest neighbors (KNN), Categorical Boosting (CatBoost), and naive Bayes (NB). The dataset was randomly split into a training set (70%) and a validation set (30%) using stratified sampling to preserve the EFG prevalence. All models were trained on the training set and evaluated on the validation set. Model performance was assessed using discrimination (area under the ROC curve, AUC; precision–recall curve, PR-AUC) and clinical utility (decision curve analysis, DCA). The optimal model was selected based on the highest AUC, with the DCA used only as an exploratory model-comparison indicator. Subsequently, SHAP (Shapley Additive Explanations) was then used to compute mean absolute SHAP values of each feature (20 amino acids + 12 confounding factors), which allowed quantifying and ranking the importance of features.

Key amino acids were defined as the top four amino acids by mean absolute SHAP value among those that met both of the following criteria: (1) ranking high in the SHAP-based feature importance ranking; and (2) being significantly associated with EFG in logistic regression Model 3. SHAP values indicate feature importance only and do not indicate the direction of association. Sensitivity analyses were performed to determine the strength of these associations and investigate the possibility of nonlinear relationships. To begin with, stratified analyses were conducted based on sex, age group, school type, MVPA status, hypertension, general obesity, central obesity, and total energy intake. For total energy intake, participants were stratified using the age- and sex-specific median of total energy intake within each subgroup. Conditional logistic regression models were used in each stratum to assess the relationships between key amino acids and EFG risk. Also, *p*-values of interaction were calculated using interaction terms and likelihood ratio tests to determine possible effect modifiers. Generalized additive model (GAM) with smoothing splines was used to assess nonlinear dose–response relationships between the intake of the key amino acids and EFG risk. The optimal smoothing parameter was determined via generalized cross-validation (GCV), and the effective degrees of freedom (edf) were reported to indicate the degree of nonlinearity. All above models were covariate-adjusted to covariates in Model 3.

R software (version 4.3.3) was used to conduct statistical analyses. MatchIt, Boruta, stats, forestplot, car, e1071, class, lightgbm, catboost, xgboost, pROC, dcurves, and mgcv were used as the following packages: propensity score matching, feature selection, basic statistical analysis, forest plot visualization, multicollinearity diagnostics, machine learning model construction (SVM, NB, KNN, LightGBM, CatBoost, and XGBoost), ROC and PR curves, DCA, and GAM. A two-sided *p*-value < 0.05 was considered statistically significant.

## 3 Results

After applying the inclusion and exclusion criteria, 12,567 participants were included in the study. PSM was conducted to reduce possible confounding bias, which left a final analytic sample of 6,960 participants, 1,740 in the EFG group and 5,220 in the normal control group (Figure 1). The selection and validation of features with the Boruta algorithm revealed that all amino acid intake-related variables were found to be significant features that were related to EFG (Figure 2).

**Figure 1:**
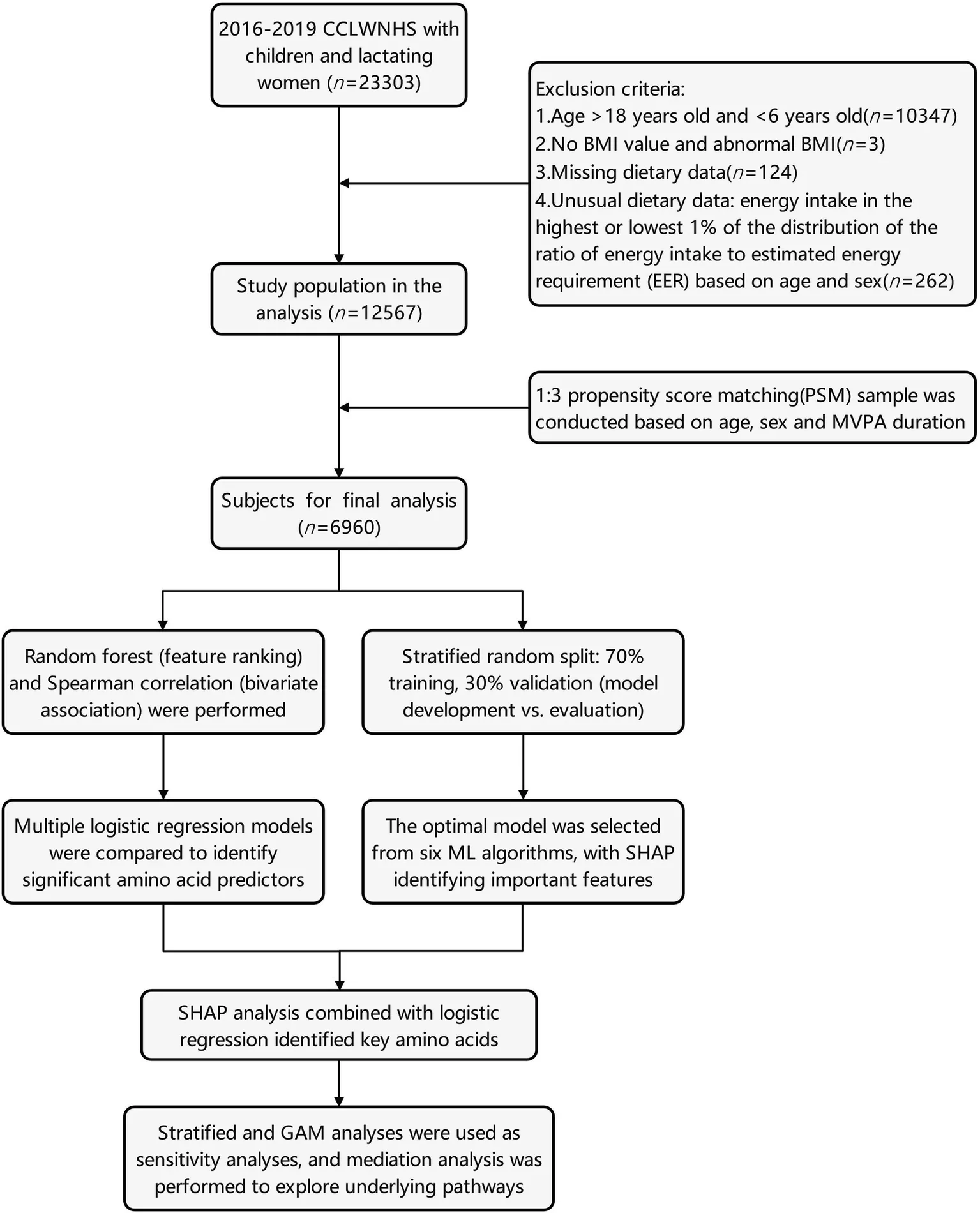
Flow diagram of study population enrollment and analytical workflow.

**Figure 2:**
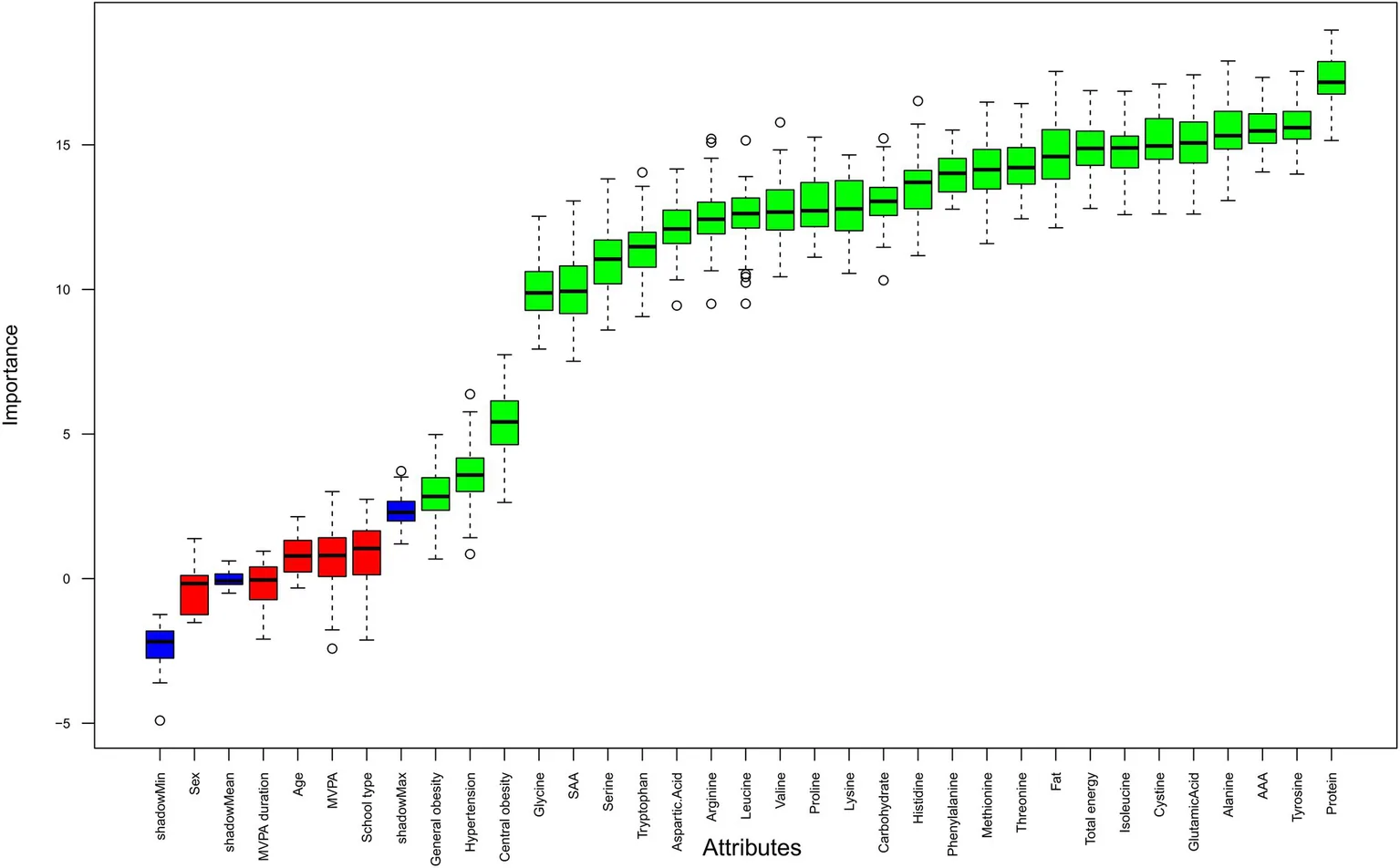
Boruta feature importance plot for variables associated with EFG risk.

Table 1 indicates that there were no significant differences between the two groups in terms of sex, age, MVPA status, or school type (*p* > 0.05). The prevalence of hypertension, general obesity, and central obesity, however, was much greater in the EFG group compared to the control group (*p* < 0.01). In terms of amino acid intake, the EFG group had much lower isoleucine, cystine, tyrosine, phenylalanine, arginine, glutamic acid, tryptophan, and total aromatic amino acid levels than the control group, but leucine, lysine, threonine, alanine, and methionine levels were significantly higher (*p* < 0.05). Food analysis also revealed that the protein, fat, carbohydrate, and total energy intakes were much greater in the EFG group compared to the control group (*p* < 0.01). Correlation analysis identified 12 dietary amino acids significantly associated with EFG in the overall population (Figure 3).

**Table 1:** Demographic and baseline features of the study population.

Variables	Total (*n* = 6,960)	Normal control group (*n* = 5,220)	EFG group (*n* = 1740)	Statistic	*p*
Sex, *n* (%)				χ^2^ = 0.00	1.000
Female	2,664 (38.28)	1998 (38.28)	666 (38.28)		
Male	4,296 (61.72)	3,222 (61.72)	1,074 (61.72)		
Age (years)	12.00 (10.00, 13.00)	12.00 (10.00, 13.00)	12.00 (10.00, 13.00)	Z = −0.25	0.804
MVPA status, *n* (%)				χ^2^ = 0.22	0.638
No	1,238 (17.79)	935 (17.91)	303 (17.41)		
Yes	5,722 (82.21)	4,285 (82.09)	1,437 (82.59)		
MVPA duration (min)	40.00 (20.00, 60.00)	40.00 (20.00, 60.00)	40.00 (20.00, 60.00)	Z = −0.34	0.736
School type, *n* (%)				χ^2^ = 2.50	0.114
Primary school	3,718 (53.42)	2,817 (53.97)	901 (51.78)		
Secondary school	3,242 (46.58)	2,403 (46.03)	839 (48.22)		
Hypertension, *n* (%)				χ^2^ = 36.48	<0.001
No	5,177 (74.38)	3,978 (76.21)	1,199 (68.91)		
Yes	1783 (25.62)	1,242 (23.79)	541 (31.09)		
General obesity, *n* (%)				χ^2^ = 33.28	<0.001
No	5,033 (72.31)	3,868 (74.10)	1,165 (66.95)		
Yes	1927 (27.69)	1,352 (25.90)	575 (33.05)		
Central obesity, *n* (%)				χ^2^ = 47.50	<0.001
No	5,635 (80.96)	4,324 (82.84)	1,311 (75.34)		
Yes	1,325 (19.04)	896 (17.16)	429 (24.66)		
Isoleucine (%)	3.62 (3.39, 3.90)	3.64 (3.40, 3.91)	3.58 (3.38, 3.86)	Z = −4.31	<0.001
Leucine (%)	7.20 (7.04, 7.41)	7.20 (7.04, 7.41)	7.22 (7.06, 7.44)	Z = −2.84	0.005
Lysine (%)	4.69 (4.26, 5.14)	4.68 (4.25, 5.13)	4.74 (4.31, 5.16)	Z = −3.03	0.002
Serine (%)	4.29 (4.18, 4.43)	4.29 (4.18, 4.43)	4.29 (4.18, 4.42)	Z = −0.39	0.694
Cystine (%)	1.26 (1.10, 1.43)	1.27 (1.11, 1.43)	1.23 (1.08, 1.40)	Z = −4.38	<0.001
Tyrosine (%)	3.21 (3.03, 3.45)	3.22 (3.03, 3.46)	3.18 (3.03, 3.38)	Z = −4.31	<0.001
Phenylalanine (%)	4.40 (4.27, 4.54)	4.41 (4.28, 4.55)	4.38 (4.25, 4.52)	Z = −4.48	<0.001
Threonine (%)	3.73 (3.54, 3.90)	3.72 (3.53, 3.89)	3.77 (3.57, 3.93)	Z = −6.20	<0.001
Glycine (%)	4.64 (4.35, 4.91)	4.64 (4.35, 4.91)	4.66 (4.36, 4.91)	Z = −0.52	0.606
Valine (%)	4.85 (4.67, 5.00)	4.85 (4.67, 5.00)	4.86 (4.68, 5.01)	Z = −1.13	0.257
Arginine (%)	5.63 (5.35, 5.94)	5.63 (5.36, 5.96)	5.60 (5.32, 5.87)	Z = −4.07	<0.001
Histidine (%)	1.79 (1.62, 1.96)	1.79 (1.62, 1.96)	1.79 (1.60, 1.95)	Z = −0.94	0.347
Alanine (%)	5.75 (5.28, 6.21)	5.73 (5.27, 6.19)	5.80 (5.32, 6.27)	Z = −3.44	<0.001
Aspartic Acid (%)	7.93 (7.52, 8.27)	7.93 (7.53, 8.28)	7.91 (7.51, 8.23)	Z = −1.64	0.101
Glutamic Acid (%)	15.69 (14.03, 17.86)	15.74 (14.08, 17.91)	15.46 (13.83, 17.71)	Z = −3.43	<0.001
Methionine (%)	2.03 (1.88, 2.17)	2.02 (1.87, 2.16)	2.07 (1.91, 2.19)	Z = −6.23	<0.001
Proline (%)	5.07 (4.48, 5.84)	5.07 (4.48, 5.84)	5.07 (4.47, 5.85)	Z = −0.16	0.873
Tryptophan (%)	1.31 (1.21, 1.39)	1.32 (1.21, 1.40)	1.31 (1.21, 1.38)	Z = −3.21	0.001
Sulfur-containing amino acids (%)	3.28 (3.12, 3.44)	3.28 (3.12, 3.44)	3.28 (3.12, 3.46)	Z = −1.32	0.186
Aromatic amino acids (%)	7.60 (7.34, 7.95)	7.62 (7.35, 7.98)	7.56 (7.33, 7.87)	Z = −4.91	<0.001
Protein intake (g)	107.01 (74.87, 150.30)	103.46 (72.40, 146.05)	116.34 (85.10, 161.09)	Z = −8.25	<0.001
Fat intake (g)	34.18 (20.97, 55.91)	32.83 (19.94, 53.92)	37.78 (24.35, 61.89)	Z = −7.70	<0.001
Carbohydrate intake (g)	314.94 (228.57, 433.89)	308.21 (225.76, 427.55)	331.42 (238.86, 459.52)	Z = −4.89	<0.001
Total energy (kcal)	2040.44 (1482.08, 2824.28)	1993.49 (1452.02, 2758.88)	2167.78 (1594.65, 2998.96)	Z = −6.91	<0.001

**Figure 3:**
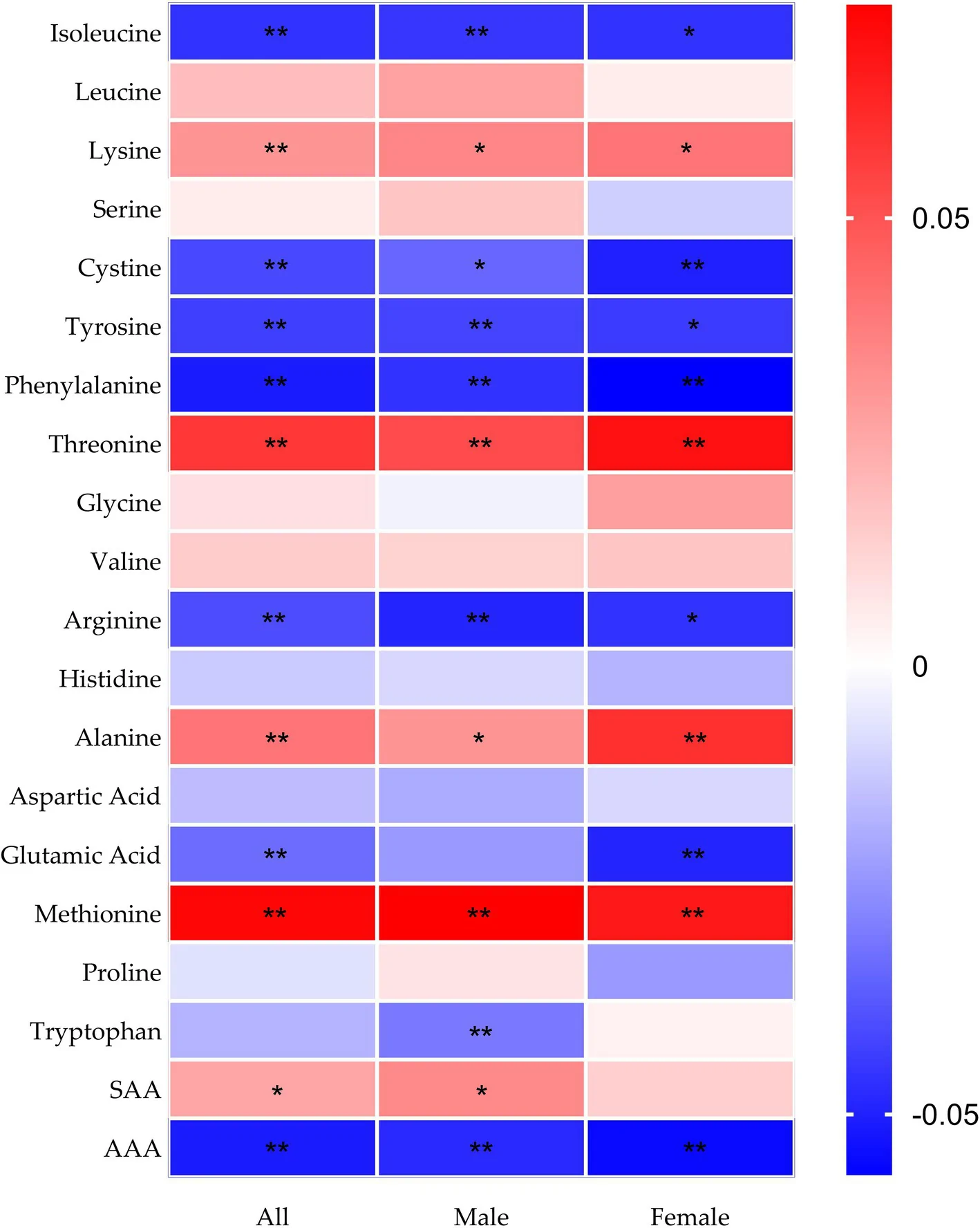
Spearman correlation heatmaps between EFG and dietary amino acids. Red: positive; blue: negative. *p < 0.05, **p < 0.01.

Prediction models of EFG in children and adolescents were built using six machine learning models (XGBoost, LightGBM, SVM, KNN, CatBoost, and NB). Among the six models, LightGBM achieved the highest validation AUC (0.831, 95% CI: 0.817–0.844) with a small training–validation AUC difference (ΔAUC = 0.043). This superior discriminative ability was further supported by the PR curve, where LightGBM consistently outperformed the other models across all recall levels. DCA additionally showed that LightGBM had the most favorable net benefit in the matched sample. Thus, the LightGBM model was chosen to be further analyzed in terms of SHAP-based feature contribution analysis (Figure 4). The contribution of 20 amino acids and 12 possible confounding factors to the mean absolute SHAP were quantified and ranked. In the SHAP-based feature importance ranking, the top-ranked amino acids were methionine, arginine, threonine, tryptophan, tyrosine, and alanine (Figure 5).

**Figure 4:**
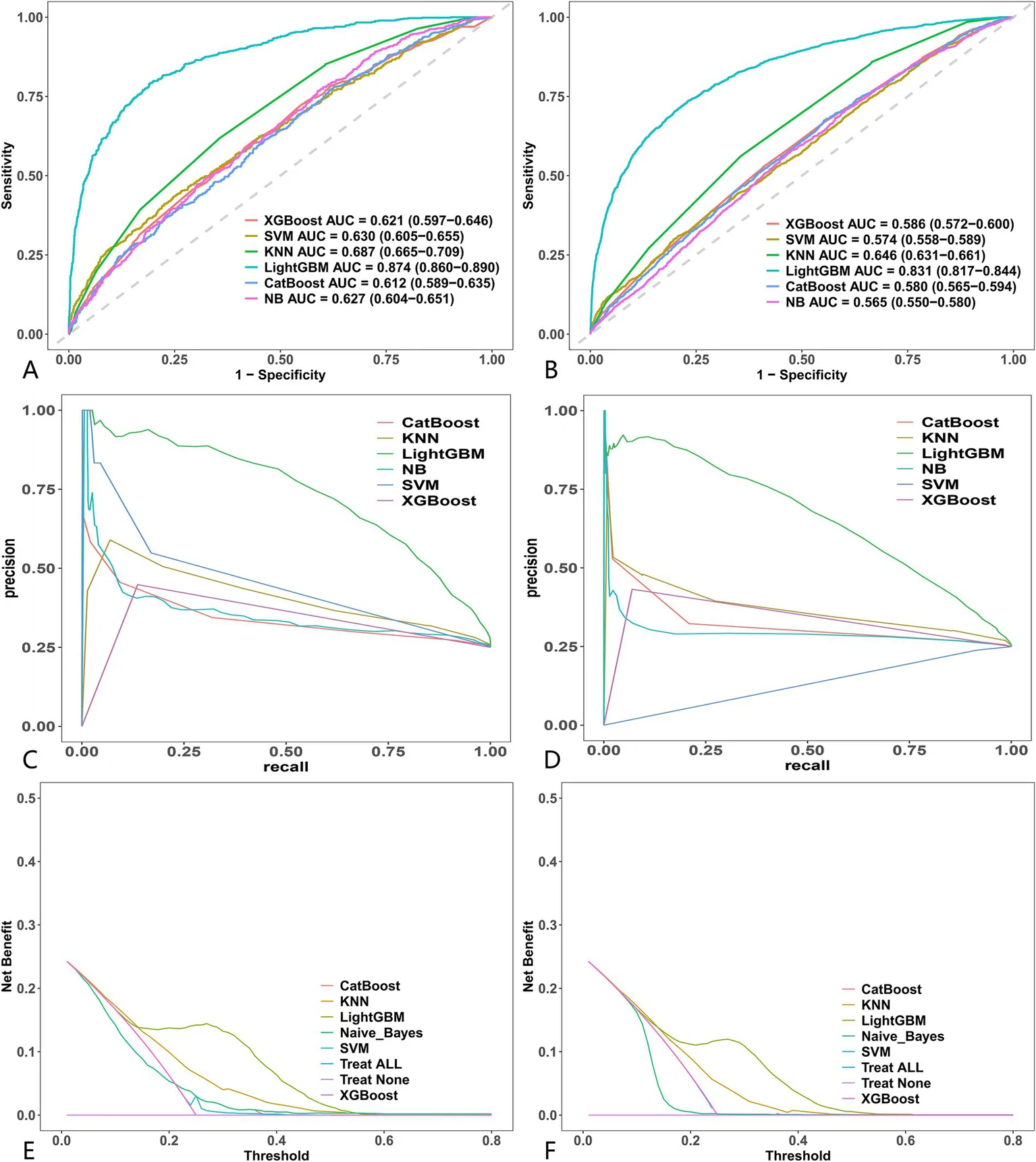
Performance evaluation of six machine learning predictive models. **(A)** ROC curves on the training set; **(B)** ROC curves on the validation set; **(C)** PR curves on the training set; **(D)** PR curves on the validation set; **(E)** DCA curves on the training set; **(F)** DCA curves on the validation set.

**Figure 5:**
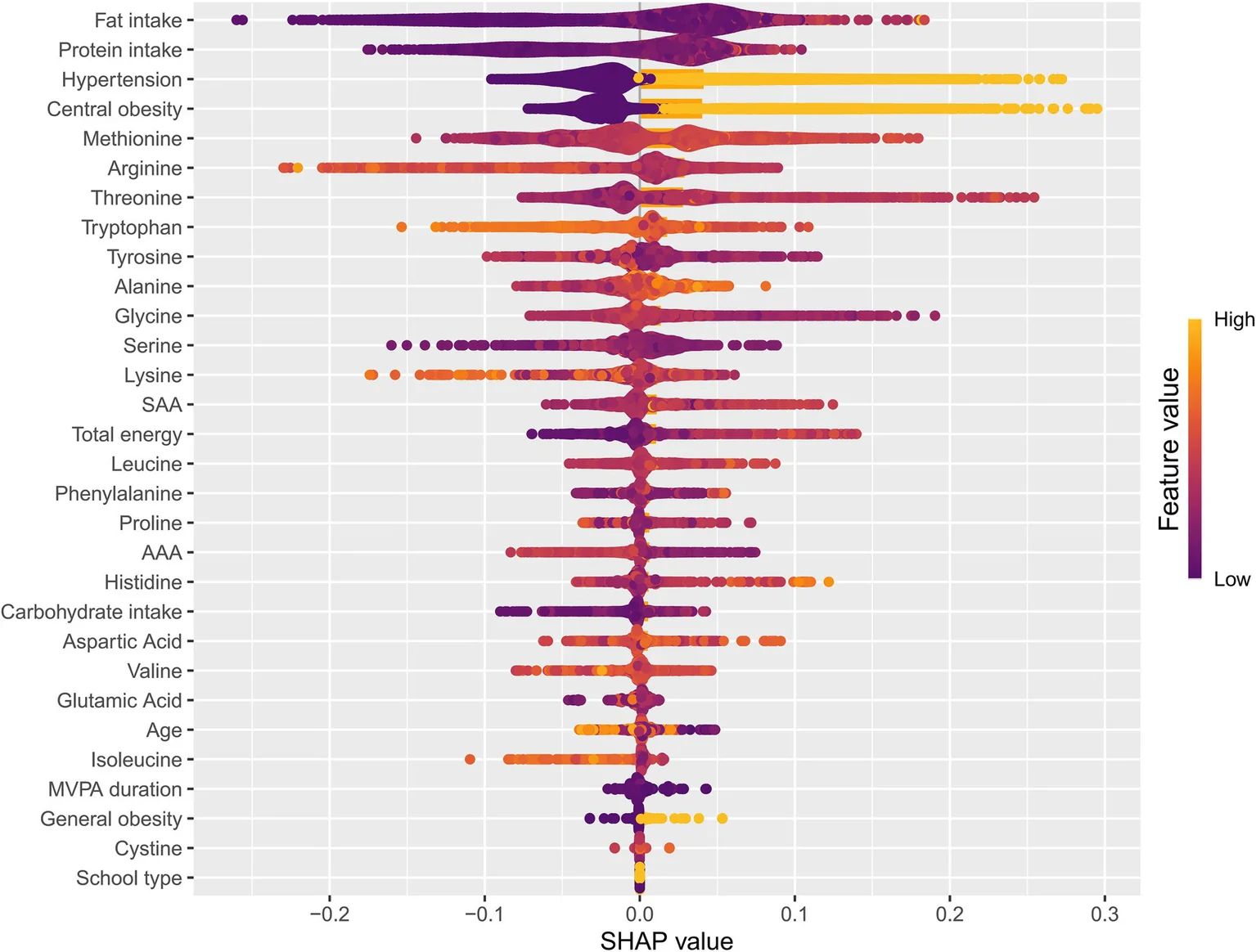
SHAP-based evaluation and interpretation of the LightGBM model. Features are ranked by mean absolute SHAP values; color represents feature value (red: high, blue: low).

Conditional logistic regression analysis showed that lysine, threonine, alanine, and methionine were positively associated with EFG risk (all *p* < 0.01). Inversely associated amino acids included isoleucine, cystine, tyrosine, phenylalanine, arginine, glutamic acid, and aromatic amino acids. The direction and strength of these associations were generally similar in all models. Only in Models 2 and 3, sulfur-containing amino acids were significant (*p* < 0.05). Leucine and tryptophan were significantly associated with EFG in the unadjusted model, but neither remained significant after adjustment. The rest of the amino acids did not show any significant associations (Table 2).

**Table 2:** Impact of different dietary amino acids on EFG risk in Chinese children and adolescents.

Amino acids	Model 1 OR (95% CI)	*p*	Model 2 OR (95% CI)	*p*	Model 3 OR (95% CI)	*p*	E-value (CI)
Isoleucine	0.73 (0.63–0.85)	<0.001	0.73 (0.63–0.85)	<0.001	0.74 (0.64–0.86)	<0.001	2.05 (1.59)
Leucine	1.21 (1.05–1.41)	0.010	1.15 (0.99–1.33)	0.069	1.13 (0.97–1.31)	0.115	1.51 (1.00)
Lysine	1.13 (1.05–1.23)	0.002	1.12 (1.04–1.22)	0.004	1.11 (1.03–1.20)	0.009	1.64 (1.19)
Serine	1.14 (0.89–1.45)	0.296	1.06 (0.83–1.36)	0.622	1.06 (0.83–1.36)	0.632	1.32 (1.00)
Cystine	0.65 (0.52–0.81)	<0.001	0.66 (0.53–0.82)	<0.001	0.68 (0.54–0.85)	0.001	2.32 (1.64)
Tyrosine	0.66 (0.55–0.79)	<0.001	0.69 (0.57–0.83)	<0.001	0.71 (0.59–0.86)	<0.001	2.16 (1.62)
Phenylalanine	0.57 (0.44–0.75)	<0.001	0.54 (0.41–0.71)	<0.001	0.56 (0.43–0.74)	<0.001	2.96 (2.06)
Threonine	1.71 (1.42–2.06)	<0.001	1.62 (1.35–1.95)	<0.001	1.59 (1.32–1.92)	<0.001	2.56 (1.97)
Glycine	0.96 (0.86–1.08)	0.498	1.05 (0.93–1.18)	0.438	1.04 (0.93–1.17)	0.467	1.26 (1.00)
Valine	1.19 (0.98–1.45)	0.085	1.14 (0.94–1.39)	0.189	1.14 (0.93–1.38)	0.206	1.53 (1.00)
Arginine	0.77 (0.69–0.86)	<0.001	0.81 (0.73–0.91)	<0.001	0.82 (0.74–0.92)	0.001	1.73 (1.39)
Histidine	0.95 (0.77–1.16)	0.603	0.90 (0.74–1.11)	0.325	0.90 (0.73–1.10)	0.307	1.44 (1.00)
Alanine	1.13 (1.05–1.23)	0.002	1.15 (1.06–1.24)	0.001	1.14 (1.06–1.24)	0.001	1.55 (1.30)
Aspartic acid	0.94 (0.87–1.02)	0.158	0.95 (0.87–1.03)	0.176	0.95 (0.87–1.03)	0.202	1.21 (1.00)
Glutamic acid	0.98 (0.96–0.99)	0.003	0.98 (0.96–0.99)	0.005	0.98 (0.96–0.99)	0.007	1.18 (1.09)
Methionine	2.00 (1.59–2.52)	<0.001	2.09 (1.65–2.63)	<0.001	2.03 (1.61–2.57)	<0.001	3.48 (2.60)
Proline	0.99 (0.94–1.04)	0.672	0.99 (0.94–1.04)	0.666	0.99 (0.94–1.04)	0.565	1.23 (1.00)
Tryptophan	0.66 (0.47–0.93)	0.017	0.74 (0.52–1.04)	0.086	0.77 (0.54–1.09)	0.141	1.40 (1.00)
Sulfur-containing amino acids	1.18 (0.98–1.43)	0.088	1.23 (1.01–1.49)	0.037	1.23 (1.02–1.50)	0.034	1.77 (1.14)
Aromatic amino acids	0.74 (0.66–0.84)	<0.001	0.75 (0.66–0.85)	<0.001	0.77 (0.68–0.87)	<0.001	1.91 (1.58)

Model 1 unadjusted. Model 2 was adjusted for total energy (continuous), school type (primary school or secondary school). Model 3 was further adjusted for general obesity (yes or no), central obesity (yes or no), Hypertension (yes or no). All models used conditional logistic regression stratified by matched set. Age, sex, and MVPA duration were matching variables and were therefore not included as covariates. The models did not estimate an intercept.

Combining the essential amino acid characteristics determined by the LightGBM model with the outcomes of logistic regression, the key amino acids were identified as methionine, arginine, threonine, and tyrosine. Methionine had the greatest model contribution and displayed a significant positive association with EFG (OR = 2.03, *p* < 0.01). Arginine and tyrosine were inversely associated with EFG (OR = 0.82 and 0.71, respectively; all *p* < 0.01), and threonine was positively associated with EFG (OR = 1.59, *p* < 0.01). The E-values for the four key amino acids were high, further verifying the robustness of their independent associations with EFG risk against potential unmeasured confounding (Table 2).

Stratified analyses revealed that the direction of associations was generally consistent across subgroups, but statistical significance varied. The interaction between methionine and EFG risk was more apparent in the group that achieved MVPA (*p* = 0.017). The protective effect of arginine appeared stronger in the low energy intake group (*p* = 0.010). Similarly, the protective effect of tyrosine was more pronounced in the non-central obesity group and low energy intake group (*p* = 0.045 and 0.023, respectively). Other stratification factors did not show any significant interactions (all *p* > 0.05; Figure 6).

**Figure 6:**
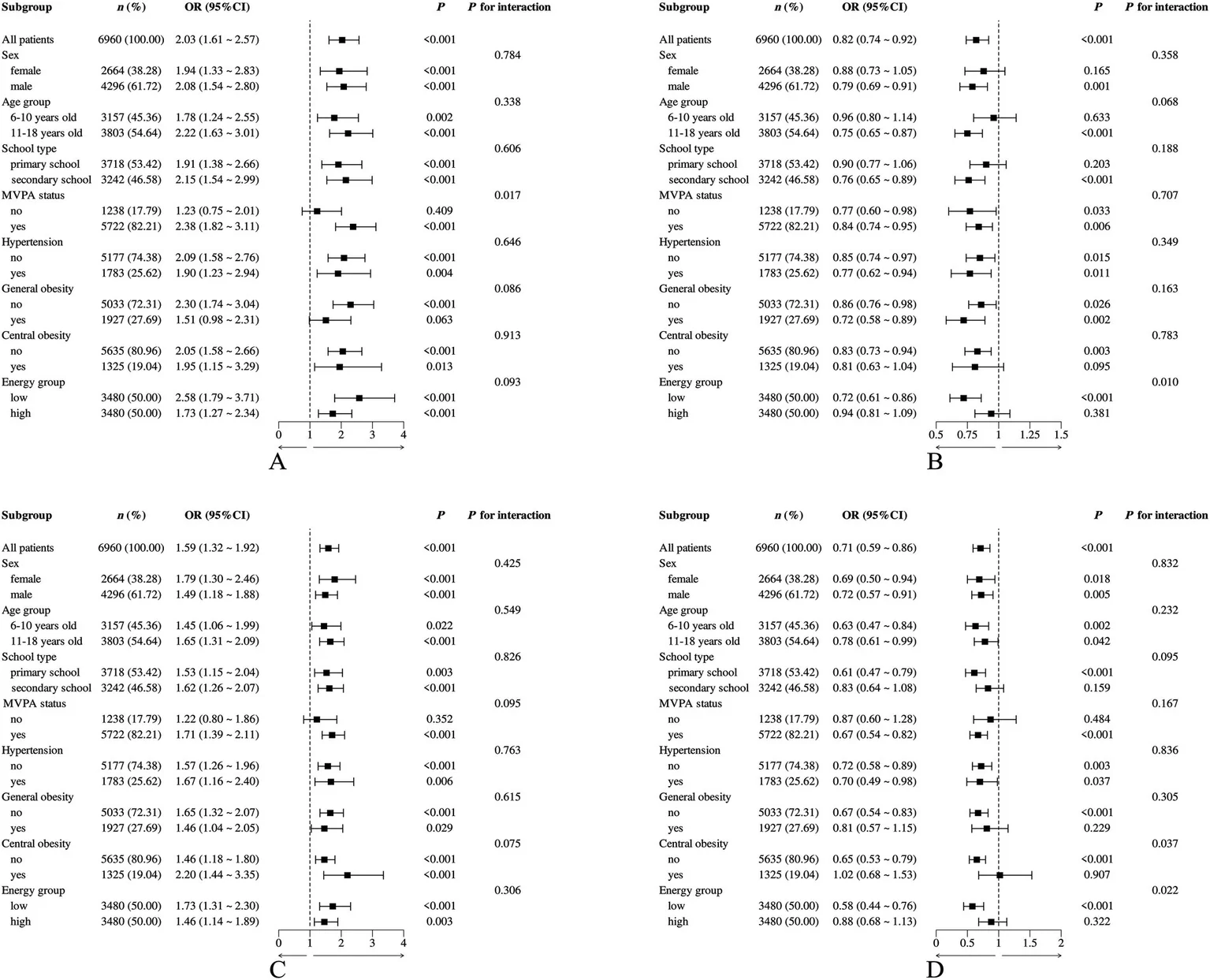
Stratified analysis of key dietary amino acids and EFG risk: a forest plot. Amino acid species: **(A)** Methionine; **(B)** Arginine; **(C)** Threonine; **(D)** Tyrosine. Stratification was performed according to the following variables: Sex (male or female), Age (6–10 years old or 11–18 years old), school type (primary school or secondary school), MVPA status (no or yes), general obesity (no or yes), central obesity (no or yes), hypertension (no or yes), total energy (low or high, dichotomized at the age- and sex-specific median).

GAM analysis revealed significant nonlinear associations for methionine (edf = 3.103), arginine (edf = 5.005), and tyrosine (edf = 4.507), whereas threonine showed a near-linear relationship (edf = 1.011; Figure 7).

**Figure 7:**
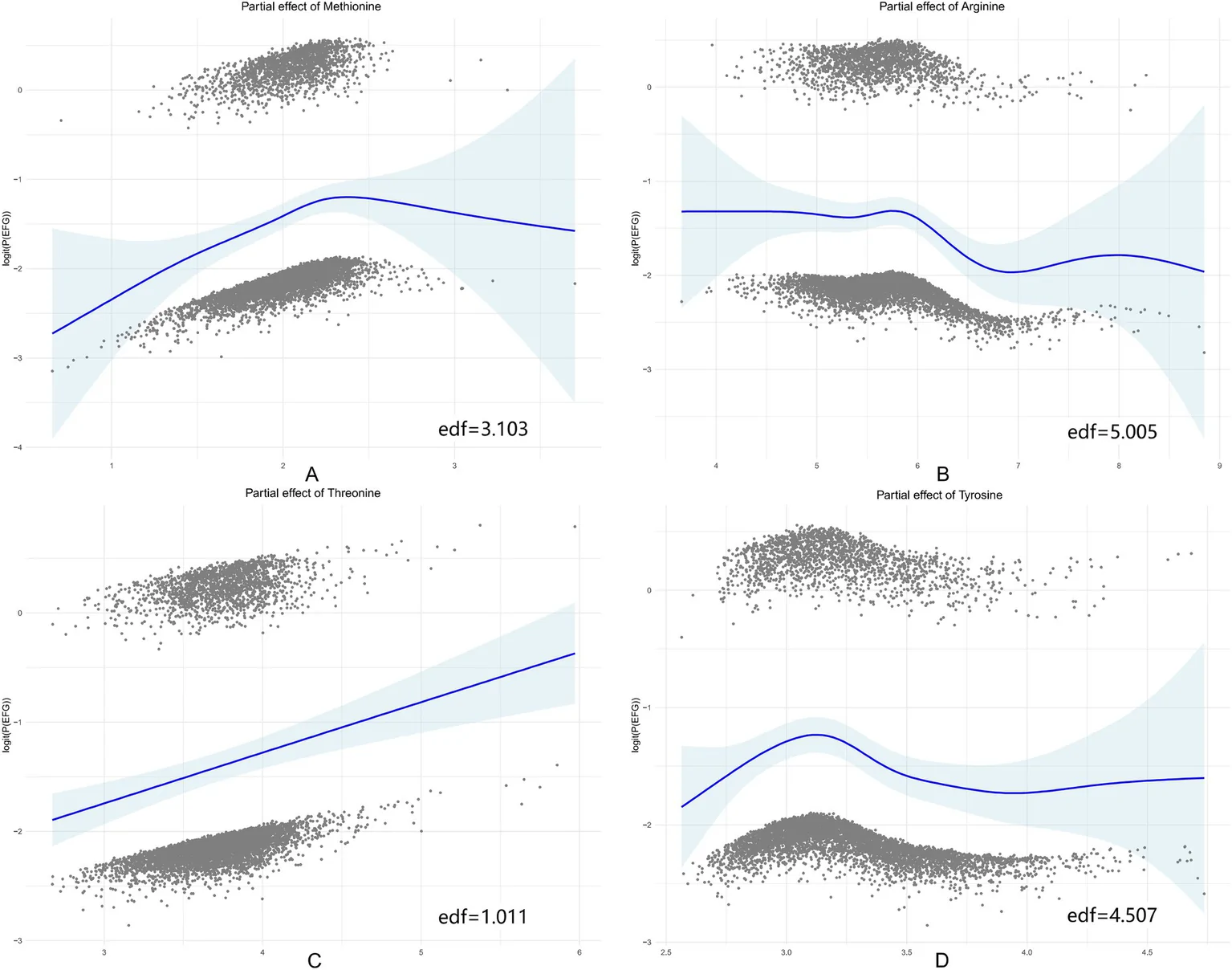
GAM-based nonlinear dose–response relationships between key amino acid intakes and EFG risk. Amino acid species: **(A)** Methionine; **(B)** Arginine; **(C)** Threonine; **(D)** Tyrosine. Partial effect plots show the smoothed relationship (blue line) with 95% confidence intervals (shaded area) on the scale of the linear predictor (logit(P(EFG))). Rug plots along the x-axis indicate the distribution of observations.

## 4 Discussion

This study systematically screened and validated four dietary amino acids: methionine, arginine, threonine, and tyrosine, which were significantly associated with EFG in Chinese children and adolescents based on data from four provinces. The findings revealed that methionine and threonine were positively associated with EFG, and arginine and tyrosine were inversely associated, all of which may be important dietary correlates associated with EFG risk in children and adolescents.

In this study, we used an integrated analytical framework that combined traditional logistic regression with machine learning–based feature ranking. SHAP was used to prioritize amino acids and narrow the candidate list, while logistic regression was used to determine the direction and statistical significance of associations. Using this integrated approach, we identified four key amino acids associated with EFG. Methionine was the most significant variable in both analysis techniques, with the largest effect size and the most important feature, which supports its positive association with EFG. This indicates that methionine might be a major amino acid target that affects glucose homeostasis in this group. This observation is in line with the large-scale epidemiological studies in China (36, 37). In animal and mechanistic studies, methionine restriction (MR) has been shown to lower blood glucose levels and improve insulin sensitivity (38–40). Mechanistically, high methionine intake may stimulate the mTORC1 signaling pathway, thus inhibiting insulin signaling and reducing insulin sensitivity (41). Moreover, excessive methionine intake increases homocysteine production, which may cause hyperhomocysteinemia and impair pancreatic β-cell function (42, 43). In contrast, MR has been reported to protect pancreatic function by alleviating endoplasmic reticulum stress and improving redox balance (44). It also stimulates hepatic secretion of fibroblast growth factor 21 (FGF-21), which can enhance insulin secretion and increase insulin signaling in the liver, adipose tissue, and skeletal muscle (39, 45, 46). In addition, MR has been shown to trigger autophagy in major metabolic organs and to regulate H₂S and DsbA-L expression in inguinal white adipose tissue (iWAT), leading to elevated adiponectin levels, increased skeletal muscle glucose uptake, and improved insulin resistance (41, 44, 47). MR also reverses the metabolic imbalances caused by excessive methionine by more widespread systemic action. First, it may enhance lipid metabolism and reduce ectopic lipid accumulation in the liver (41, 48–50). Second, it alters gut microbiota composition by increasing short-chain fatty acid–producing bacteria, thereby improving insulin sensitivity (42, 51). Third, it also may reduce oxidative stress and NF-κB–mediated chronic inflammation (41, 47, 52). Together, these processes may provide a holistic pathophysiological explanation of methionine-related dysregulation of glucose homeostasis.

Threonine was also found to be a risk-related amino acid of EFG in this study. In Chinese populations, epidemiological research has revealed that increased dietary threonine consumption is strongly linked with the risk of developing T2DM (36). Moreover, hyperglycemia or IR patients have increased plasma threonine levels (53, 54). These results align with the findings of the current study, which further suggest a possible harmful effect of threonine in the pathogenesis of T2DM. The pathways through which threonine can interfere with glucose homeostasis are probably multiple. First, being a glucogenic amino acid, threonine can be converted in the liver through the dehydrogenase/aldolase and dehydratase pathways to glucose precursors, including pyruvate and 2-oxobutanoate, which directly enter the gluconeogenesis pathway and thus lead to elevated blood glucose levels (12). Second, being an essential amino acid, excess threonine can overactivate the mTORC1, which can disrupt insulin signaling transduction, induce IR, and eventually result in high blood glucose levels (12, 55). Moreover, a large-scale population-based study conducted in China has also found a positive correlation between threonine consumption and the risk of obesity (36). Animal studies have also shown that threonine restriction can greatly decrease body weight and fat mass in mice (56), which indicates that excess threonine consumption can be a cause of obesity and systemic metabolic imbalance. Since obesity is a proven risk factor of T2DM, it can also indirectly facilitate hyperglycemia by a series of interrelated pathophysiological mechanisms.

Conversely, arginine were highly protective. Clinical research has demonstrated that arginine can greatly lower blood glucose, regulate insulin levels, and ameliorate IR. To this end, arginine supplementation has been suggested as a possible adjunctive treatment of diabetes (57–60). The pathways may include a number of mechanisms. First, arginine enhances insulin secretion by stabilizing glucokinase (GCK) in pancreatic β-cells, thus inhibiting its ubiquitination and degradation and increasing β-cell glucose-sensing ability. Moreover, being a cationic amino acid, arginine enters β-cells and causes membrane depolarization, opens voltage-dependent calcium channels, stimulates Ca^2+^ influx, and directly stimulates insulin release (61). Second, arginine stimulates the incretin axis. Oral arginine stimulates the release of glucagon-like peptide-1 (GLP-1) by intestinal endocrine L cells, which in turn enhances insulin secretion in response to glucose and enhances glucose tolerance (60, 62). Third, arginine stimulates the AMP-activated protein kinase (AMPK) pathway. It causes AMPK expression and activation in skeletal muscle, liver, and adipose tissue, increasing glucose uptake and use in skeletal muscle and improving systemic energy metabolism (63, 64). Fourth, arginine is the only substrate of NO synthesis through nitric oxide (NO)-mediated vascular effects, which promote vasodilation, enhance blood flow to insulin-sensitive tissues, and aid the delivery of insulin and glucose. It also decreases oxidative stress caused by hyperglycemia and aids in maintaining β-cell activity (60, 64–66). Fifth, arginine enhances lipid metabolism and insulin sensitivity by lowering the level of serum triglyceride and free fatty acid, and thus, decreasing IR caused by lipotoxicity (64, 67). Lastly, arginine metabolism is also strongly connected with the composition of gut microbiota and can indirectly enhance glucose homeostasis through the generation of beneficial metabolites like short-chain fatty acids (68, 69). In this paper, dietary arginine intake was found to be inversely associated with EFG risk and its protective effect is in line with the above-described mechanisms, which further justifies the positive role of arginine in glucose homeostasis in children and adolescents.

This paper also discovered that the risk of EFG in children and adolescents was significantly negatively correlated with tyrosine intake, which indicates that it could be a significant protective factor in glucose homeostasis. Nevertheless, this observation contradicts certain earlier adult studies (10, 70, 71), and the mechanisms underlying this finding remain unclear. One possible explanation is that the dietary tyrosine may be metabolized in the intestine and then converted to dopamine (72, 73). In childhood and adolescence, the metabolic destiny of tyrosine can be more heavily oriented to protein synthesis and the production of central neurotransmitters leading to a higher level of dopamine activity in the central nervous system. This can have glucose-lowering effects by controlling appetite and feeding behavior, and by controlling energy metabolism homeostasis (73, 74). However, in adults or under pathological conditions, dopamine can be more peripheral and possibly a cause of IR or insulin secretion (73, 75). Thus, this age-related disparity in the physiological targets of dopamine action could be one of the reasons why the intake of tyrosine is negatively correlated with blood glucose in this study. However, this hypothesis requires further investigation. Another plausible explanation is that a higher proportion of tyrosine may reflect a dietary pattern with greater intake of dairy or soy protein. Dairy and soy proteins are rich in aromatic amino acids, including tyrosine. Therefore, the inverse association observed in this study may partly reflect differences in dietary protein sources, not merely a direct protective effect of tyrosine itself.

Surprisingly, GAM analysis showed that methionine, arginine, and tyrosine were all significantly nonlinearly related to EFG risk. In particular, methionine exhibited a trend of risk increment at low-to-moderate levels of intake, and a gradual decrease in the effect at higher levels of intake. This inverted U-shaped correlation could be due to the fact that, in childhood and adolescence, the body still has some level of metabolic compensatory ability to excessive intake of methionine, which could be a major metabolic difference with adults. Conversely, the protective effects of both arginine and tyrosine showed a similarity of rapid decrease beyond an inflection point, indicating that there was a threshold to the beneficial effect of these two amino acids. When the intake is low, the amino acids are first used in the basic metabolic processes and their protective effects are yet to be fully realized. At intake ratios above the threshold, arginine and tyrosine can better engage in glucose regulatory pathways, resulting in a rapid development of protective effects. These nonlinear characteristics have important public health implications. In children and adolescents, it is not about the fact that the lower the intake of risk-related amino acids, the better, or the higher the intake of protective amino acids, the better. Rather, it is important to focus on the appropriate proportions of amino acids intake. The future studies need to elaborate on the best intake levels and the critical levels of these amino acids to give a quantitative foundation on the development of dietary guidelines.

There are a number of strengths in this study. First, it is an innovative combination of conventional epidemiological techniques and machine learning algorithms, which enhances the accuracy and strength of the key target identification. Second, the exposure measure was the nutrient density method (i.e., the ratio of individual amino acids to the total protein intake), which is more effective in reducing the confounding effect of total protein intake and is more representative of the quality of amino acid intake than the quantity of amino acid intake.

However, this study also has several limitations. To begin with, dietary intake was measured by FFQ, which is prone to inherent recall bias and measurement error. In addition, cooking oil and condiments were measured at the household or canteen level and allocated to individuals in proportion to energy intake, which may underestimate individual fat intake. Amino acids were expressed as percentages of total protein intake, so compositional and food-source confounding cannot be excluded. Furthermore, objective validation was not done using serum amino acid biomarkers, which can undermine the validity of exposure assessment. Second, genetic predisposition, gut microbiota composition, sleep status, family history of diabetes, pubertal stage, socioeconomic status, sugar-sweetened beverage intake, and overall diet quality were not considered or had substantial missingness, which can alter the relationship between amino acid metabolism and glucose metabolism, and may lead to residual confounding. Third, the analysis was restricted to four of the 31 provinces participating in the parent surveillance. These provinces cover eastern, northern, and southwestern China but do not represent all regions of China. In addition, survey weights and cluster identifiers were not available for this four-province subset, so the complex sampling design was not incorporated. Fourth, the propensity-matched sample fixed EFG prevalence at 25%, discarded a substantial number of participants, and may retain residual confounding because matching was performed only on age, sex, and MVPA duration. Fifth, fasting plasma glucose was measured only once, which may not fully capture long-term glucose status. In addition, this study was a cross-sectional observational study, which can only determine the associations between amino acid intake and EFG but not causality, and reverse causation cannot be excluded. To further confirm these findings, future research must include Mendelian randomization studies or prospective cohort studies.

## 5 Conclusion

This study used a systematic screening and validation of Chinese multi-province surveillance data to identify significant associations between four dietary amino acids, methionine, arginine, threonine, and tyrosine, and the risk of EFG in children and adolescents. Methionine and threonine were found to be independent dietary correlates of EFG risk, but arginine and tyrosine were independent protective dietary correlates. These findings may inform hypothesis generation for future studies on the protein composition of the diet and nutritional recommendations in children and adolescents, and provide preliminary data for future research on amino acid-based nutritional regulation.

## Data Availability

The raw data supporting the conclusions of this article will be made available by the authors, without undue reservation.
